# Bilateral Papilledema and Right Esotropia as an Initial Presentation of Acute Myeloid Leukemia in a Young Girl

**DOI:** 10.7759/cureus.25413

**Published:** 2022-05-27

**Authors:** Reem M Hersi, Ayat M Aldosari, Nada K Naaman, Rawan K Alrajhi, Abdullah S Alqahtani

**Affiliations:** 1 Medicine, King Saud Bin Abdulaziz University for Health Sciences, Jeddah, SAU; 2 Ophthalmology, King Abdulaziz Medical City, Jeddah, Saudi Arabia, Jeddah, SAU; 3 Ophthalmology, Vitreoretinal/Ocular Oncology Surgery, King Saud Bin Abdulaziz University for Health Sciences, Jeddah, SAU

**Keywords:** pediatric oncology, anemia, intracranial pressure, leukemia, esotropia, papilledema

## Abstract

Leukemia is a malignant hematologic neoplastic disease in which acquired mutations and genetic abnormalities in early hematopoietic precursors cause rapid proliferation of white blood cells (WBC). Acute myeloid leukemia (AML), a subtype of leukemia, is a rare form of cancer that typically manifests in adulthood. Symptoms typically arise due to abnormal proliferation of WBC. Ocular manifestations of such malignancies are rare and they occur more commonly in acute lymphoblastic leukemia (ALL) rather than AML. Furthermore, ophthalmic involvement usually is either a sign of central nervous system involvement or disease relapse. In this article, we report the case of a 14-year-old girl who presented initially with double vision and right eye squint. The patient was later diagnosed with AML with leptomeningeal involvement.

## Introduction

Leukemia is a malignant hematologic neoplastic disease in which acquired mutations and genetic abnormalities in early hematopoietic precursors cause rapid proliferation of white blood cells (WBC) [1,2]. Acute myeloid leukemia (AML), a type of leukemia that typically manifests in adulthood, arises due to the accumulation of immature cells of the myeloid lineage in the bone marrow and peripheral blood [3]. AML patients commonly present with anemia, bruising, bleeding gums, and organomegaly. A bone marrow aspiration is used to make a definitive diagnosis [3]. Ocular manifestations of leukemia are caused by either direct infiltration of neoplastic cells or indirectly by hematological abnormalities, opportunistic infections, or as a complication of treatment [4,5]. It is estimated that ocular involvement occurs in up to 90% of cases, either as the disease's initial manifestation or as a sign of relapse [4,6,7]. In this article, we present a case of a 14-year-old girl who initially presented with right eye squinting, double vision, and headache. The patient was later diagnosed with AML with leptomeningeal involvement.

## Case presentation

**Figure 1:**
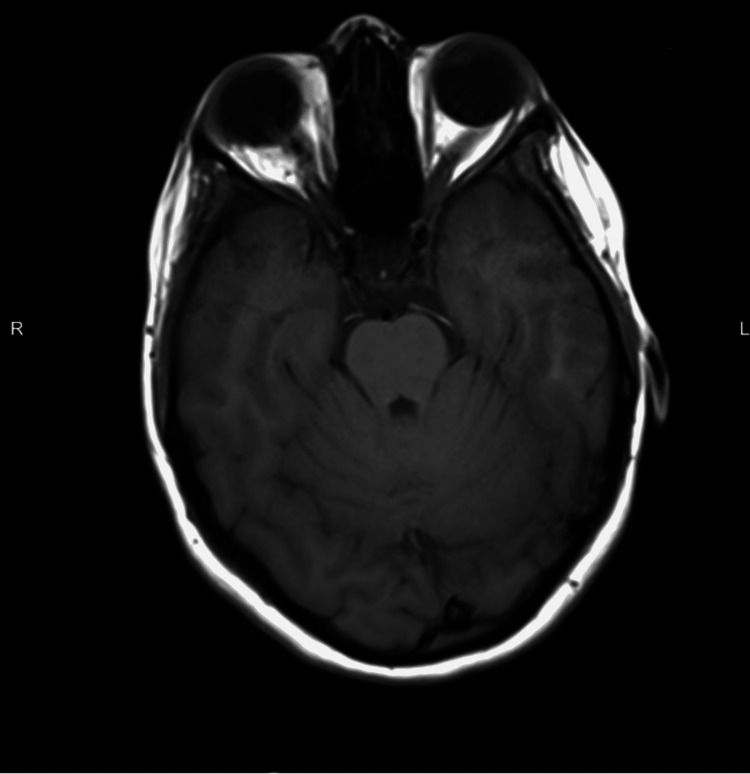
Axial section of brain MRI showing leptomeningeal enhancement

A 14-year-old girl was referred from another hospital after presenting with a double vision of the right eye for one and a half months. Headache and bleeding gums were two other complaints. She had diplopia in the right gaze, a minor right eye esotropia, and a right eye limitation in abduction. The intraocular pressure was 14 mmHg bilaterally, and the visual acuity, based on Snellen Chart, was 6/12 bilaterally. The examination of the anterior segment was unremarkable. An examination of the fundus revealed moderate to severe bilateral optic disc edema with no signs of retinal or choroidal infiltrates. Further examination revealed that the patient had no facial palsy, no lymphadenopathy, no organomegaly, or central nervous system (CNS) focal deficits. The patient had five healthy siblings and no personal or family history of hematological disease or malignancy. Complete blood count (CBC) showed: hemoglobin 6.5 g/dl, mean corpuscular volume 97.4 fl, mean corpuscular hemoglobin 33.5 pg, hematocrit of 18.9%, red blood cell 1.9 x1012/L, WBC 11.3 x109/L, and platelets 52 x109/L. A peripheral blood smear revealed 80% blasts, while a peripheral blood flow cytometry revealed 47% blasts. Based on the available positive work-up, the patient was admitted as a case of newly diagnosed acute leukemia to be investigated further. Increased Intracranial pressure (ICP) was suspected; therefore, a CT scan of the brain was done and revealed arachnoid granulations in the left transverse sinus resulting in stenosis and increased (ICP). To rule out leptomeningeal involvement, a brain MRI was performed (Figure 1).

**Figure 2:**
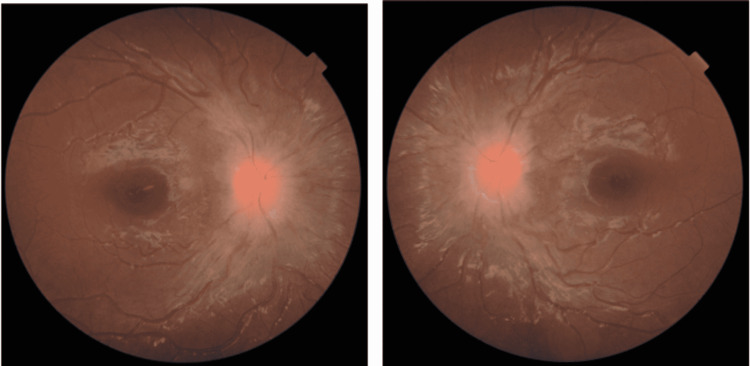
Fundus photographs showing bilateral papilledema without infiltration

The MRI revealed diffuse supratentorial and infratentorial leptomeningeal enhancement, as well as involvement of the right abducens nerve, which is consistent with leptomeningeal disease. Fundus photographs were obtained revealing bilateral hyperemic optic disc swelling with blurred disc margin and no infiltration (Figure 2).

Flow cytometry analysis of peripheral blood sample showed 39% cell population expressing CD19, CD33, CD 34, CD117, and HLA-DR negative for CD7 and CD10. Morphological assessment revealed Auer rods in the blasts. In addition, Flow cytometry analysis of bone marrow aspirate showed cell population in the blast region constituting around 31% of all nucleated cells and expressing CD11, CD13, CD15, CD19, CD33, CD34, CD117, and CD123. The aforementioned results were consistent with AML. Karyotyping revealed a t(8;21) translocation, and molecular testing showed the expression of Wilms' tumor 1 (WT1). The patient was treated initially with fludarabine, cytarabine, and granulocyte-colony stimulating factor with idarubicin (FLAG-Ida) as induction therapy, in addition to three doses of intrathecal chemotherapy with methotrexate/cytarabine. Acetazolamide and topiramate were used to treat the high ICP. During induction, the patient was afebrile and clinically stable. Cytological analysis of the bone marrow showed 0.8% blasts post induction. On the four-week follow-up after the diagnosis, visual acuity was 6/12 OD (right eye) and 6/7.5 OS (left eye) with a resolution of papilledema. Long-term follow-up is not available as the patient was lost to follow-up with Ophthalmology. Consent to publish this article had been obtained. 

## Discussion

Ocular involvement is less common in patients with acute leukemia than in those with chronic leukemia [8]. Ocular presentation as a sign of relapse of acute leukemia has been well documented, in acute lymphoblastic leukemia (ALL) more often than AML [9,10]. The occurrence of ocular manifestations at the time of diagnosis, however, is not frequently encountered. Initially presenting as a CNS-localized disease, with signs of optic nerve involvement and increased ICP, this is a rare case of newly diagnosed AML.

Ocular involvement may manifest in any part of the eye, including the optic nerve, retina, choroid, and anterior segment [4]. Multiple papers studied the prevalence of ocular manifestation in leukemia patients before initiation of treatment. Karesh et al. reported retinopathy in 50% of AML patients [11]. Reddy et al. [12] and Alemayehu et al. [13] observed ocular findings in 35% and 32% of leukemia patients, respectively. Bitirgen et al. found ocular involvement in 36% of AML patients [14]. Initial presentation with ocular involvement was also reported by individual case reports in different types of leukemia [15,16].

Involvement of the optic nerve can occur directly as a result of leukemic cell infiltration or indirectly as a result of hematological abnormalities and disturbed hemostasis [17]. The optic nerve protects leukemic cells from pharmacological treatments, making it the culprit behind relapse in most cases [18]. In a study by Robb et al., six out of 60 children dying of acute leukemia had optic nerve involvement. Five of the six patients had coexisting intracranial meningeal involvement, indicating that the optic nerve involvement in acute leukemia is closely related with CNS and meningeal disease [19]. This was also the case in an acute promyelocytic leukemia patient with optic nerve involvement who was found to have leukemic infiltration of the leptomeninges [20]. This link highlights the impact that ocular manifestations have on prognosis, especially when the optic nerve is involved. 

This case also presented with signs of increased ICP including bilateral papilledema and right sixth cranial nerve palsy. The mechanism behind the intracranial hypertension is perhaps the infiltration of blasts across the arachnoid granulations, altering cerebrospinal fluid reabsorption. This is supported by a few reported cases of leukemic patients with increased ICP [5,15].

## Conclusions

In conclusion, the presented case highlights the possibility of AML presenting with ocular involvement at diagnosis. To the best of our knowledge, this is one of the rare cases in which an AML patient presents initially with ocular involvement. Therefore, although uncommon, AML should be in the differential diagnosis of children presenting with ocular manifestations such as visual field changes, especially if accompanied by systemic symptoms. With early detection and guided laboratory evaluation, vision can be saved if therapy is initiated promptly.
